# Observations on Dr. Cayley's Case of Aneurism

**Published:** 1807-05

**Authors:** 


### *To the Editors of the Medical and Physical Journal.*

They who judge unjustly, most commonly do it from ignorance or malice.

GENTLEMEN,

EVERY man is not endowed with the genius of writing with that logical fluency and propriety, which may be called delightful, and which arrests the attention whatever is the subject. Your Journal exhibits many instances of this; while, on the other hand, many useful and practical observations are committed to the eyes of your readers in language not so very elegant.

In a work like yours, every paper is liable to a critical examination, and it is what every one has to expect; no medical diploma can screen him from it, and that too from the meanest apothecary who may be able to expose the fallacy of his reasoning, or detect error. These were my thoughts upon perusing some of the papers in your 95th Number; but I mean to confine my present Observations to Dr. Cayley's paper on Aneurism. The most remarkable circumstance of this case, is Mr. Strothers not being able to distinguish that it was aneurism. He healed the *deep* wound by the first intention in ten days; "Mr. S. then discontinued his visits, thinking his patient was doing well, being able to walk about, though not without pain, and some apprehension of a recurrence of hæmorrhage." How came the patient to be apprehensive of hæmorrhage when the wound was so quickly healed by the first intention? In five or six weeks Mr. S. was again called, when a considerable tumour had formed upon the part, attended with pain and constitutional irritation, spasms, and incapability of using the leg. It had been poulticed before Mr. Strother came. What did he order them to do? "To foment the part every three hours with hot fomentations, and afterwards poulticed. This plan was strictly pursued for a *long time*, without producing any change that appeared favourable; the skin became a deep red, and appeared rather thinner than in its healthy state. Mr. Strother now resolved, that if the tumour should not break, he would at his next visit lay it open. On his arrival he proceeded to examine it, but to his great surprise and *mortification* he found it to be an aneurism, and told his patient his opinion, and the probable result; which gave Mr. W. considerable uneasiness, as he dreaded the operation, and was was fearful that the artery should burst, and carry him off."

Could it be possible for any surgeon, who had ever heard of aneurism, or knew the pulsatory power of the arterial system, to have mistaken the diagnosis of aneurism so far, as to treat it as a common abscess, when Dr. C. observes, "that the sac was of a considerable size, the pulsation through the whole was not only tangible but visible across the room? Dr. C. surely means to expose the discernment of Mr. Strother, and place it in the most conspicuous point of view, whose practice in general ought to have been kept in the back ground; it however paves the way for what follows, when he brings Mr. Stubbs forward. But before I enter upon the defence of Mr. Stubbs, I have Dr. Cayley's successful method of cure to examine. He considers his practice of compression as a novel mode of treatment; but if he peruses any Treatise of Surgery, he will find that it is a practice which has been tried many times with various success, when the aneurism was formed on a small branch of an artery; when the artery is of a lage size, it is in vain to attempt such a palliative mode of treatment.

On the 6th of September, when Messrs. S. and C. met to examine the result of Dr. C's new mode of treatment, "the tumour was completely removed; inflammation very trifling, and not the slightest pulsation; the bandage was again applied, and the ancle to be rubbed with the volatile liniment, and to be moved in different directions."— It is mentioned that during the course of the ten or twelve days compression, "that sometimes there was pain, sometimes none at all; at one time considerable numbness, with a prickling sensation was felt in the whole limb, from the posterior part of the thigh to the toes, which he could not move, nor his ancle joint." This appears to me to have been a very anomalous Aneurism, which could not be distinguished at first by Mr. C. from abscess, and now had symptoms of wounded nerve and muscular irritation. This is more to be remarked, when it is said on the 6th of September, that the tumor was completely removed, and again on the 22d, when it is said, "on the part where the wound was inflicted, there was a small colourless elevation, without the slightest pain; this I considered as a mere thickening of the integuments; *it was perfectly firm to the touch.*" I am at a loss what to think of this, but more so, when some of the patient's friends had thought it necessary to ask the the opinion of another practitioner; although Dr. C. assured Mr. W. that he had *nothing to apprehend.* Mr. Stubbs had certainly seen something more to warrant his advising the application of caustics. I should be glad if he would communicate what he observed through the medium of your useful Journal; the medical public calls for it, as well as the unwarranted attack upon his character. It is a disgrace to the science of medicine that such difference occurs between those who practise it. They who possess but moderate talents, and a diploma obtained not by merit, but purchased by money or interest, are most clamorous for reform, and the Lord knows what. But in my opinion, it would be a means of destroying all subordination, as well as damping the spirit of emulation, and introducing a system of privileged oppression. I think there cannot be a perfect equality of talents, therefore, there must always be good and indifferent practitioners, whatever reformation take place, nor can the answering a few questions, which may be learned like the catechism, ever make a complete physician or surgeon; or finish the genius of the mind, or dexterity of the hand. Alas! we often find that the employment of practitioners does not depend upon talents.

I could draw some very curious characters, exhibiting the pride, arrogance, and ignorance of some country practitioners; but at present I must confine myself to the defence of Mr. Stubbs, whose advice in this case, did not seem to me so very absurd as Dr. C. would make it appear. For, allowing it to have been Aneurism, it was only one of the branches of the poplitea, and from the hæmorrhage being so readily stopped by the thumb, it may have been only one of the small ramifications of the tibial artery.

When a great arterial trunk is cut or wounded, it is no easy matter by compression to stop the hæmorrhage, especially where the artery lies deep; and when it is had recourse to, great swelling of the adjacent parts is the consequence.

I remember being called to a case where the ulnar artery was divided by a carpenter's chisel: It occurred in a country village, at a considerable distance from medical advice. His neighbours had succeeded in stopping the hæmorrhage, by filling the wound with cobwebs and great compression. On the second day it began again to bleed. He then came into town and had the artery sewed by a surgeon. There was now a considerable swelling upon his wrist, hand, and fingers, which extended up his arm to the the shoulder: Although the wound, when first inflicted, was not more than one inch in size; by the great swelling it was considerably enlarged. On the 4th day after it was sewed, the artery began again to bleed; the wound now looked very ill, having a gangrenous appearance; an attempt was again made to secure it, with very little hope of answering; it, however, prevented the recurrence of the hæmorrhage for three days, but on the 4th day it again returned; any attempt to sew it was now deemed improper. A little lunar caustic was applied to the part, and milled lint dipped in tinct. Benzoes comp. applied to the wound—which never bled afterwards. The wound was bathed every day with the tinct. Benzoes comp. A pledget with the ungt. basilic. flav. and the cortex peruv. taken internally, were the only applications used: and, although the patient's arm was swelled equal to the greatness of his thigh, in one month's time he was in a condition to return to his friends again. And, in twelve months after this accident, when his name was taken up, to be balloted for the Militia, he came and wanted a certificate for exemption from me; but when I examined the part, there was so little appearance of there ever being a wound upon his wrist, that I could not grant him one.

I have inserted this Case, to show the effects of compressing the artery and leaving it for some days unsecured; the swelling was very alarming of itself without the hæmorrhage, and I could only attribute it to the compression. The efficacy of the caustic is also deserving attention; but as the tinct Ben. c. was also used, I will not take upon me to say which had most share in stopping the hæmorrhage. How far Mr. Stubbs's proposal deserves such harshness and censure as Dr. C. is pleased to bestow upon him for it, I shall leave with the public to judge; I think that it has more of novelty in it than Dr. Cayley's; but could only be attempted by a person skilled a little in Surgery. I must declare, that I would have ventured to have used it in this case myself, without endangering the patient's life; and when he was not willing to undergo the operation, should have proposed it as the next practical method. I think Mr. Stubbs deserves thanks for his hint, for which I acknowledge myself indebted; and if a similar case occur in my practice, I will give Dr. Cayley's novel method a fair trial; and if I do not succeed by compression, I will next try Dr. Stubbs's.

I must in this place observe an expression of Dr. Cayley's, which is not intelligible to me, "far Doctors." Why is is Mr. Stubbs called an imposture, a pest of society? Dr. C. like diplomatic characters, should have first shown his own powers to the public, before he called Mr. Stubbs self dubbed, that we might have been the better enabled to judge of the talents of those who dubbed him. Very often a man may as well be dubbed by himself as another, and he may possess the most genius of the two. Before Dr. C. had treated Mr. S. in such a harsh manner, he should have had something more to relate of him than is here before the public. Mr. Stubbs had not erred more grossly than Mr. Strother, who is as much the subject for medical reform as Stubbs, when he treated it as a common abscess, and was going to lay it open. Wherein consisted the difference of laying it open with a lancet or caustic? The latter was certainly preferable, if it had the appearance of aneurism. Dr. C. hopes medical reform will put a stop to every new hint, or novel method of cure, but what comes from an M. D. I should embrace a useful discovery even from the meanest plebian, and think him entitled to my regard. Dr. C. may, no doubt, wish the Reform also to extend to every family, and by act of parliament prevent any one from taking a single dose of physic for himself, without the advice of an M. D.

I have encroached too much upon your attention and the patience of the readers of your Journal, I shall therefore conclude by observing, that when Dr. C. is likewise drawing to an end with his paper, and giving the reasons which induced him to try this novel method of cure, he says, "This led me to recommend continued pressure, in hopes too of bringing the wounded parts of the artery into contact, and thereby to promote *cicatrization*. This is surely a word that is improperly used here; the word implies the skinning over of a wounded external surface, or the reproduction of the cuticle. In this case, there was no wounded surface to cicatrize, the wound being healed by the first intention; I therefore think that Dr. C. has somewhat to dread from a medical reform as well as Dr. Stubbs.

CRITO.

